# Microstructured Macromaterials Based on IPN Microgels

**DOI:** 10.3390/polym13071078

**Published:** 2021-03-29

**Authors:** Irina Rashitovna Nasimova, Vladimir Yurievich Rudyak, Anton Pavlovich Doroganov, Elena Petrovna Kharitonova, Elena Yurievna Kozhunova

**Affiliations:** 1 Faculty of Physics, Lomonosov Moscow State University, 119991 Moscow, Russia; vurdizm@gmail.com (V.Y.R.); doroganov.a@mail.ru (A.P.D.); harit@polly.phys.msu.ru (E.P.K.); kozhunova@polly.phys.msu.ru (E.Y.K.); 2 Russian Academy of Science, 119991 Moscow, Russia; 3 Faculty of Chemistry, Lomonosov Moscow State University, 119991 Moscow, Russia

**Keywords:** microgels, interpenetrating networks, computer simulations, poly-N-isopropylacrylamide, polyacrylic acid, hydrogels, smart materials

## Abstract

This study investigates the formation of microstructured macromaterials from thermo- and pH-sensitive microgels based on interpenetrating networks of poly-N-isopropylacrylamide (PNIPAM) and polyacrylic acid (PAA). Macromaterials are produced as a result of the deposition of microgel particles and subsequent crosslinking of polyacrylic acid subnetworks to each other due to the formation of the anhydride bonds during annealing. Since both PNIPAM and PAA are environment-sensitive polymers, one can expect that their conformational state during material development will affect its resulting properties. Thus, the influence of conditions of preparation for annealing (pH of the solution, the temperature of preliminary drying) on the swelling behavior, pH- and thermosensitivity, and macromaterial inner structure was investigated. In parallel, the study of the effect of the relative conformations of the IPN microgel subnetworks on the formation of macromaterials was carried out by the computer simulations method. It was shown that the properties of the prepared macromaterials strongly depend both on the temperature and pH of the PNIPAM-PAA IPN microgel dispersions. This opens up new opportunities to obtain materials with pre-chosen characteristics and environmental sensitivity.

## 1. Introduction

Among polymer materials with high potential for applications, polymer gels are of particular importance. Of greatest interest in this aspect are the so-called sensitive or “smart” gels. Since “smart” gels are able to change their volume in response to changes in environmental conditions, they can be used to create systems that find their application in various practical fields: for example, in the development of drug carriers, various types of sensors, etc. [1,2,3,4,5,6]. Many of the technologies based on polymer gels require the use of microscale objects, e.g., targeted drug delivery technologies or paints production, inkjetprinting, molecular separation, environmentally sensitive display devices, and molecular switches development. This gave impetus to the development of such a direction of polymer science as the synthesis and investigation of microgels [7,8,9,10]. First of all, the advantage of microgels is that they are particles of submicron size and this leads to a faster and reversible response to changes in the external environment, in contrast to macrogels. Possible uses for microgels other than macrogels include, for example, stabilization of colloidal systems [11]. The introduction of various additives into microgels—drugs, metal nanoparticles, biopolymer molecules, dyes, significantly expands the range of areas for their application [12,13,14,15].

The desire to take advantage of microgels at the macro scale has led scientists to use microgels as building blocks to create extended two- and three-dimensional structures [16,17,18,19,20]. To obtain films and macromaterials based on microgels, various principles are used, such as adsorption or grafting on chemically modified surface [20,21,22], layer-by-layer assembly [19,23], and crosslinking of microgel particles citeGuan2011, Cai2008.

Several investigations are devoted to the study of films and coatings made of thermosensitive poly(N-isopropylacrylamide) (PNIPAM) and its copolymers, and the influence of various factors on their properties, such as the method of film production, concentration, particle size, amount of crosslinking agent [20,24,25]. The use of environment-sensitive microgels makes it possible to create on their basis microstructured macromaterials with the ability to control their pore sizes, refractive index, mechanical properties, etc. [19,26]. In addition, it can be assumed that such materials will have shorter response times to external influences as compared to traditional macrogels-based materials, which makes them promising for many areas of application. For example, studies are known in which microgels were used to create photonic crystals like structures [27]. A group led by L.A. Lyon has created sensitive microlenses from microgel ensembles based on NIPAM and acrylic acid copolymers. These microlenses can be optically switched over short time intervals [28] and used to detect proteins [29]. In addition, films and coatings made of PNIPAM microgels and their copolymers can be used to inhibit bacterial adhesion [30] or as biosensors to enzymes [31]. In the work of Litowczenkoa et al., it was proposed to use the porose PNIPAM films formed by microgel particles for possible tissue engineering applications.

**Figure 1:**
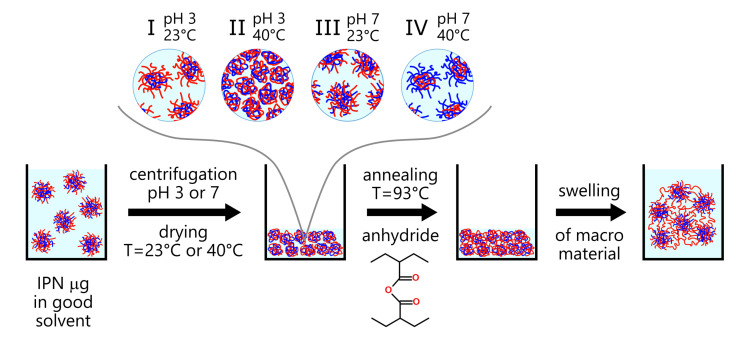
Scheme of the formation of material from microgels. IPN particles consist of two independent but topologically entangled networks: PNIPAM (red) and PAA (blue).

In this study, we are going to use microgels based on two interpenetrating networks: poly(N-isopropylacrylamide) and poly(acrylic acid) (PAA) IPN microgels [32,33] to obtain three-dimensional macromaterials on their basis as a result of the deposition of microparticles and subsequent crosslinking of polyacrylic acid networks to each other (Figure 1).

IPN microgels are a relatively new object of research, in particular, microgels based on PNIPAM and PAA. The original method for their synthesis was proposed by X. Xia and Z. Hu [34]. Since both PNIPAM and PAA are environment-sensitive polymers, microgels based on them are double sensitive and their swelling coefficient and conformational state of the subnetworks can be governed both by temperature and pH. This opens up additional possibilities for controlling the microstructure and swelling behavior of the resulting material during its formation.

Earlier [35], we proposed and tested three methods for the development of PNIPAM-PAA IPN-based macromaterials (microstructured macrogels) as a result of: (1) annealing at a temperature of 93${\;}^{\circ }$C—this treatment presumably promotes the formation of intermolecular anhydride bonds due to acrylic acid carboxyl groups; (2) crosslinking of carboxyl groups with divalent ions; (3) recrosslinking of reversible disulfide-based crosslinker (N, N’-bis-acryloyl-cystamine) incorporated in the polyacrylic acid subnetwork. It was determined that the first method (annealing at 93${\;}^{\circ }$C) is optimal in terms of the uniformity of the distribution of the resulting crosslinks. The temperature effect occurs simultaneously throughout the entire volume of the sample, while in the case of ions or disulfide-based crosslinker, local inhomogeneities in the concentrations of the introduced chemical reagents could be formed. In addition, the annealing method requires fewer manipulations with samples, therefore, it is convenient for obtaining a large sample volume. This manuscript is devoted to a more detailed study of this method of obtaining macromaterials. We are going to focus on investigating the effect of temperature and pH (let us say the quality of the solvent) in the process of development of macromaterials on their final properties.

Since in a real experiment it is difficult to avoid the influence of many other factors on the formation of macromaterials, studies were also carried out using the method of computer simulation. This will help to highlight important patterns and more rationally plan experiments to obtain macromaterials with the desired properties.

## 2. Materials and Methods

### 2.1. Materials

N-isporopylacrylamide (NIPAM—monomer, Sigma-Aldrich, Munich, Germany), Acrylic acid (AA—monomer, Sigma-Aldrich), N,N’- methylenebisacrylamide (BIS—crosslinking agent, Sigma-Aldrich), ammonium persulfate (AMPS—initiator, Sigma-Aldrich), tetramethylethylenediamine (TEMED, Sigma-Aldrich), polyacrylic acid (PAA, Mw 3,000,000, Sigma-Aldrich). Acrylic acid was purified by distillation. Other compounds were used as received. Water was purified using a Millipore Milli-Q system.

### 2.2. Synthesis of IPN Microgels

The synthesis of IPN microgels was carried out in two stages. At the first stage, parent microgels based on N-isopropylacrylamide homopolymer were synthesized. At the second stage, acrylic acid networks were synthesized inside the microgel matrices. PNIPAM homopolymer microgels were obtained by the radical thermally initiated precipitation polymerization of NIPAM in an aqueous solution in the presence of the crosslinker (the BIS concentration was 1 mol.% in terms of the monomer). The monomer concentration in the reaction mixture was 1 wt.%; the initiator AMPS concentration—0.07 wt.%. The polymerization occurred in a nitrogen atmosphere at a temperature of 70${\;}^{\circ }$C under continuous magnetically stirring at a rate of 800 rpm for 24 h. The aqueous dispersions of the synthesized microgels were gradually cooled to room temperature and purified by dialysis (the dialysis bags pore size ∼ 20,000 kDa). The synthesis acrylic acid network was carried out by radical redox-initiated polymerization under the conditions where the microgel matrices occurred in the swollen state. It was performed as follows: a 0.1 wt.% dispersion of PNIPAM microgels was prepared in deionized water, to which the BIS crosslinker, ammonium persulfate initiator, and TEMED were added. The polymerization occurred in a nitrogen atmosphere at a temperature of 23${\;}^{\circ }$C under continuous magnetically stirring at a rate of 800 rpm for 120 min. The aqueous dispersions of the synthesized IPN microgels were purified by dialysis (the dialysis bag pore size ∼ 20,000 kDa) to remove low molecular weight compounds and unreacted monomer.

### 2.3. Macromaterial Preparation

The procedure for the preparation of macromaterials from IPN microgels was the following: the dispersions of PNIPAM-PAA microgels were dried at different conditions and then annealed at a high temperature. The annealing promotes the formation of intermolecular anhydride bonds due to the dehydration of the carboxyl groups of the acrylic acid, and, accordingly, the interparticle crosslinking of the IPNs. The sample preparation process consisted of four stages (see also Figure 1):

(1) The aqueous dispersions of PNIPAM-PAA IPN microgels were concentrated by centrifugation and removal of the supernatant. It was experimentally found that for the formation of stable material, it is required to obtain solutions with a concentration of at least 25 g/L. At a lower concentration, incomplete crosslinking of particles with each other leads to the destruction of the macromaterial during swelling.

(2) The pH of the concentrated solutions was adjusted to 3 and 7 by adding NaOH and H_2_SO_4_. Depending on the acidity of the medium, the carboxyl groups of acrylic acid can be protonated and deprotonated (pKa ∼4.8), which influences both the conformation of PAA subchains and the possibility of anhydride formation.

(3) The resulting concentrated dispersions were dried at a temperature of T = 23${\;}^{\circ }$C or 40${\;}^{\circ }$C (below and above PNIPAM LCST). The need for this stage is caused by the fact that annealing without preliminary drying does not lead to the formation of high-quality macro objects. Apparently, this is due to too intense evaporation of a large amount of water simultaneously with the crosslinking process.

(4) The dried films were annealed in an oven at 93${\;}^{\circ }$C for 2 days. Then the samples were placed in distilled water and left to swell to an equilibrium state. The obtained materials showed high stability and did not dissolve for at least 6 months.

Model macromaterials from linear polyacrylic acid were prepared using the following procedure. PAA with $M_{w}=3\times 10^{6}$ g/mol was dissolved in water in concentration of 20 g/L. Then four samples of PAA solution with varying pH values (equal to 3, 5.5, 7, and 11) were prepared by addition of appropriate amounts of either 1M/L H_2_SO_4_ or 1M/L NaOH. After equilibration, the samples were dried at temperatures of 23, 40, and 93${\;}^{\circ }$C. The time for complete drying was 14, 5, and 2 days, respectively.

### 2.4. Instrumental Methods

Fourier-transform infrared spectra (FTIR) were measured on a Bruker VERTEX 70 spectrometer (Bruker, Germany) in the wavelength range 4000–500 cm${}^{-1}$. The samples were preliminarily ground into powder in an agate mortar and pressed into KBr tablets weighing 0.2 g. The weight of the test substance in the tablet was 0.005 g.

Scanning electron microscopy (SEM) images were obtained using a Prisma E microscope (Thermo Scientific, Brno, Czech) with 10 kV and 20 kV accelerating voltage in the high vacuum mode. The sample preparation procedure was the following. The pieces of PNIPAM-PAA macromaterials with a size of about 1×1×0.2 cm were placed in a mixture of water and tret-butanol (to avoid the formation of ice crystals), then frozen and slowly dried under vacuum to preserve the inner structure. Directly before the SEM measurements, the samples were dipped in liquid nitrogen and chipped. Then they were placed on conductive carbon tape and coated with 10 nm layer of gold by Q150R ES plus sputter coater (Quorum Technologies, East Sussex, UK).

Gravimetrical measurements were performed with the use of Ohaus Pioneer balance.

### 2.5. Computer Simulations

The structures of IPN microgels were prepared by simulating the two-stage radical polymerization process in the framework of coarse-grained (CG) molecular dynamics (MD) method with implemented “mesoscale chemistry” [36]. In the first stage, we prepared the primary networks by the same protocol as in Ref. [37]. The primary network of 8889 particles contained 98.5% monomers, 0.5% initiators, and 1% crosslinkers. In the second stage, IPN microgel were prepared using the same protocol as in Ref. [36]. For this, the primary networks were diluted to 1.1% mass concentration and equilibrated in a good solvent, then the components of the secondary network were added up to 2.4% total mass concentration. Monomer:crosslinker:initiator ratio of 97.5:2.0:0.5 was used for secondary networks. The system was equilibrated in a “good” solvent. Then we ran the secondary network radical polymerization. It resulted in the formation of IPN microgel with conversion rate $c_{\mathrm{IPN}}=N_{b}/N_{b}^{\max}>0.999$, where $N_{b}$ is a number of bonds created during polymerization process, $N_{b}^{\max}$ is a maximum possible number of bonds in the system. When the polymerization was finished, the sol fraction was removed, and the resulting structures were equilibrated in a “good” solvent. The resulting IPN microgels consisted of 17,635 particles (mass fraction of the first and the second networks were 50.4% and 49.6%, correspondingly).

To simulate the preparation of macrogels from microgels, the following procedure was used. First, a large simulation box with a diluted solution of 125 IPN microgel particles ($2.2\times 10^{6}$ particles, 0.5% mass concentration) was generated. It was equilibrated in three types of solvents (good, poor, and selective) for $1\times 10^{6}$ CG MD steps. Then these systems were compressed to 90% mass concentration during $1\times 10^{6}$ more CG MD steps to simulate the drying process. Next, the crosslinking of these systems into a macrogel was simulated by implementing PAA monomer–monomer bonding. For this, monomers of the first network were assigned with valence equal to one, defining the maximum number of additional bonds it can form. The reaction between monomers was implemented probabilistically. At regular time intervals (200 CG MD steps), the distance between pairs of monomers particle with free valencies was compared to the reaction radius $R_{\mathrm{react}}=1$. Monomers with a bond distance of 1 and 2 were discarded from the crosslinking procedure. An additional bond was formed with the probability $p_{\mathrm{react}}=0.001$. The reaction was ran until the conversion rates $c_{\mathrm{macro}}$ of 0.3% (∼$1.2\times 10^{3}$ CG MD steps), 1% (∼$3.8\times 10^{3}$ CG MD steps), 3% (∼$1.3\times 10^{4}$ CG MD steps), and 10% (∼$4.3\times 10^{4}$ CG MD steps). The conversion rate $c_{\mathrm{macro}}$ was defined as the ratio between the number of the additional bonds formed and half the number of monomers of the first network. Then, each system was equilibrated for $1\times 10^{6}$ CG MD steps.

In all simulations, the same implicit solvent model in NVT ensemble was used, as in previous works [36,37]. In this model, the solvent was presented via the interactions between particles instead of addition of the specific solvent particles. Nose–Hoover thermostat with temperature T=1 with temperature damping time $\tau_{dump}=50$ was used. Cutoff 12/6 Lennard–Jones potential with $\varepsilon_{LJ}=2$ and $\sigma_{LJ}=1$ was applied to all pairs of particles. The solvent quality was controlled by Lennard–Jones potential cut-off radii between particles of the first network ($R_{cut}^{\left(1\right)}$) and the second network ($R_{cut}^{\left(2\right)}$). Lower values resulted in polymer swelling, while higher values resulted in collapse (see Figure 3 in Ref. [37] for details). The cut-off radius of interaction between particles of the first and the second networks $R_{cut}^{\left(1-2\right)}$ represented the ability of these networks to have the attraction between the composing polymers relatively to the solvation (e.g., to form hydrogen bonds). Various solvent conditions were simulated as follows: “good” solvent—$R_{cut}^{\left(1\right)}=R_{cut}^{\left(2\right)}=R_{cut}^{\left(1-2\right)}=1.12$, “poor” solvent—$R_{cut}^{\left(1\right)}=R_{cut}^{\left(2\right)}=R_{cut}^{\left(1-2\right)}=3$, “selective” solvent—$R_{cut}^{\left(1\right)}=1.12$, $R_{cut}^{\left(2\right)}=3.0,R_{cut}^{\left(1-2\right)}=1.4$. For all bonds, FENE potential with bond strength $K_{b}=30$, equilibrium bond length $R_{b}=1.5$, and LJ parameter $\varepsilon_{b}=1$ were used. Harmonic angle potential with angle strength $K_{a}=1$ and equilibrium angle $\theta_{a}=180^{\circ }$ was used. All simulations were performed in LAMMPS package [38].

## 3. Results and Discussions

### 3.1. Experimental Study of Macrostructured Materials from IPN Microgels

As discussed in the introduction, in this work, we focused on the development and study of the properties of macromaterials formed as a result of the deposition and subsequent crosslinking of microgels based on two interpenetrating networks of poly(N-isopropylacrylamide) and poly(acrylic acid). The crosslinking occurs only between the monomeric units of the acrylic acid. Both PNIPAM and PAA are environment-sensitive polymers: PNIPAM is thermosensitive and polyacrylic acid is pH-sensitive. That is, the conformation of individual subchains and microparticles volume as a whole depends on both temperature and pH [32]. The PNIPAM network undergoes a transition from a swollen to a collapsed state at temperatures above 32${\;}^{\circ }$C. For a PAA network, depending on the acidity of the medium, carboxyl groups can be protonated or deprotonated (pKa ∼4.8) and the effect of polyelectrolyte swelling could be observed at high pH. Since the resulting macromaterial will consist of two components sensitive to changes in temperature and pH, one can expect that both its swelling behavior and internal structure will depend on these two factors. In addition, it can be expected that the structure and properties of the resulting macromaterial will depend on the pH and temperature of the microgel particles dispersions during their deposition and preparation for the annealing.

**Figure 2:**
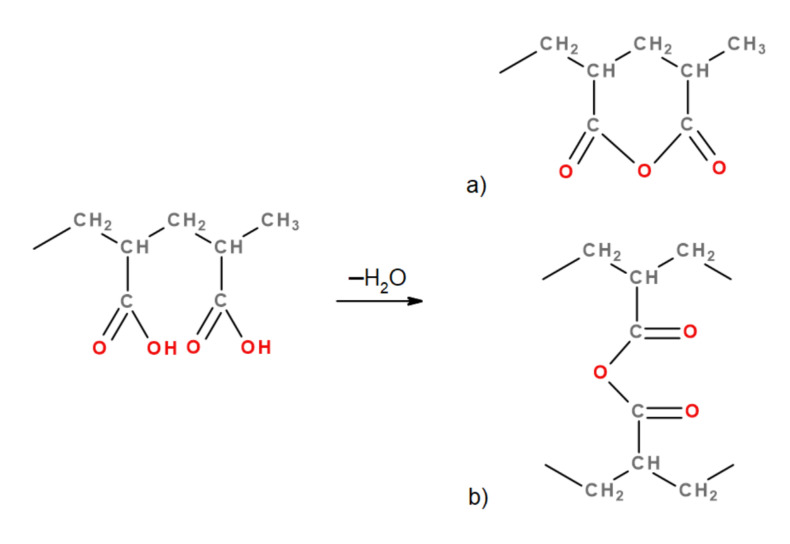
The reaction of the formation of polyacrylic acid anhydride with the formation of (**a**) intramolecular and (**b**) intermolecular structures.

Annealing at a temperature of 93${\;}^{\circ }$C was chosen as a method for interchain crosslinking of the polyacrylic acid subnetwork of IPN microgels into a single percolating macronetwork. It is known that such treatment promotes the formation of the anhydrides O=RC−O−RC=O as a result of degradation of carboxyl groups [39] COOH (Figure 2). These anhydride crosslinks can be formed between AA units in one polymer chain, within the subchains of one network, and between the neighboring microgel particles. Only the latter can contribute to the formation of stable macrogel.

To study the influence of the conformation of both microgel subnetworks during their crosslinking on the properties of the resulting macromaterial, the pH of the dispersions was controlled to be 3 and 7, and the concentrated dispersions were dried at temperatures of 23${\;}^{\circ }$C (room temperature) and 40${\;}^{\circ }$C before the annealing. The combination of these parameters makes it possible to realize the four different conformations of IPN structure: (I) pH 3, $T=23{\;}^{\circ }$C—PAA network in a shrunken state, PNIPAM network in a swollen state; (II) pH 3, $T=40{\;}^{\circ }$C—both networks in a shrunken/collapsed state; (III) pH 7, $T=23{\;}^{\circ }$C—both networks in a swollen state; (IV) pH 7, $T=40{\;}^{\circ }$C—PAA network in a swollen state, PNIPAM network in a collapsed state (see Figure 1). Let us consider how the conditions of preparation for annealing (relative conformations of IPNs) affect the properties of the resulting materials.

**Figure 3:**
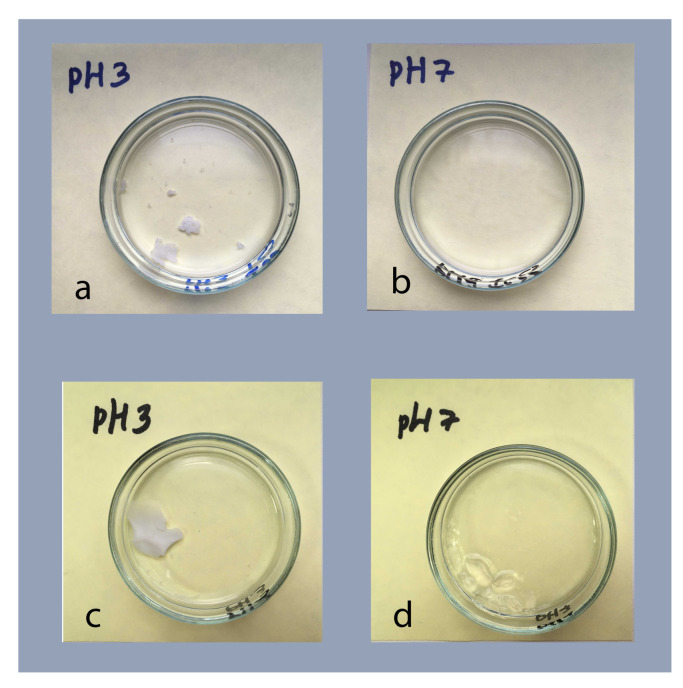
Photographs of macromaterials based on IPN microgels obtained by annealing from concentrated dispersions dried at 23${\;}^{\circ }$C (**a**,**b**) and at 40${\;}^{\circ }$C (**c**,**d**), and at pH 3 (**a**,**c**) and 7 (**b**,**d**).

All the chosen parameters allow to form the stable macrosized materials, see Figure 3 for the photographs of the four obtained macrogels samples. Yet, it could be noted that its exterior depends strongly on the preparations conditions and could be divided into two types. For both drying temperatures, the materials formed at pH 3 are dense and opaque, which may indicate the presence of inner inhomogeneities of the order of the wavelength of visible light. The materials formed at pH 7 are much more swollen and transparent, resembling the conventional homogeneous macrogels synthesized on the basis of NIPAM or AA in good solvent conditions.

The comparative studies of the swelling coefficients of all the samples confirm the visual observations (see Table 1). The degree of swelling of the macromaterials obtained from dispersions with pH 7 is an order of magnitude higher than for films obtained from dispersions with pH 3, both for samples preliminary dried at room temperature and for samples dried at T = 40${\;}^{\circ }$C. Since it is known that the swelling degree of polymer gels depends on the number of crosslinks, it can be assumed that at high pH, when the carboxylic groups are dissociated and PAA is in a swollen state, the less dense percolated macronetwork is formed during the annealing. With respect to the comparison of $m_{\mathrm{sw}}$/$m_{\mathrm{dry}}$ for films obtained at the same pH, but pre-dried at different temperatures (PNIPAM subnetwork are in the swollen or collapsed state), it can be seen that for films at pH 3 (PAA network is in the shrunken state) the swelling coefficient is higher for films pre-dried at T = 40${\;}^{\circ }$C.

Now let us discuss the influence of preparation conditions on the pH- and thermosensitive properties of the obtained macromaterials. Swelling coefficients $m_{T=45}$/$m_{T=25}$ and $m_{\mathrm{pH}\;8}$/$m_{\mathrm{pH}\;3}$ are presented at Table 1 and characterize the response of the materials to the changes in solvent quality. Interestingly, the films obtained at pH 7 have very low thermal sensitivity, which is surprising for PNIPAM-based macrogels. Swelling coefficient $m_{T=45}$/$m_{T=25}$ is about 0.9 for both samples dried at T = 23 and 40${\;}^{\circ }$C, while for conventional PNIPAM macrogels this ratio is about 0.4, meaning that the microstructured macromaterials almost do not contract while heated above PNIPAM LCST. It might be probably due to the fact that during the films preparation the PAA subnetworks are in a swollen state and the crosslinks form mainly between the acrylic acid groups outside the PNIPAM subnetworks of IPN microgel particles. Thus, as the temperature rises the PNIPAM microsubnetworks collapse, while the AA macronetwork matrix remains mostly swollen and holds the gel volume.

The analysis of $m_{\mathrm{pH}\;8}$/$m_{\mathrm{pH}\;3}$ showed that pH sensitivity of the macromaterials depends both on preparation pH and temperature. Some materials strongly react to the changes in pH of the solutions, while others almost do not swell or contract. However, a trend is difficult to establish. It should be noted that the macromaterials obtained at pH 3 and pre-dried at room temperature turned out to be quite tough and brittle and almost did not respond to changes in the external environment, such as pH and temperature. Possibly, these effects are associated with a high percentage of crosslinking of PAA subnetworks within individual microgels, which fixes the structure of the entire particle.

Thus, the conditions from which the PNIPAM-PAA IPN microgel macromaterials were obtained strongly affect their properties. So far, we have only discussed it in terms of subnetwork microgel conformations; however, in addition, both temperature and pH can influence the reaction of the formation of anhydride bonds between the carboxyl groups of poly(acrylic acid) and, therefore, the resulting degree of crosslinking of PAA macronetwork.

For a more detailed investigation of this issue, the model studies were carried out on pure PAA to exclude the effect of PNIPAM on the interpretation of the results. Preliminary tests showed that pure PNIPAM—either linear or in the form of microgel particles—does not allow the formation of stable materials using the chosen treatment, thus it does not participate in the formation of crosslinks. The model PAA macromaterials were prepared from linear PAA solutions of different pH and drying/annealing temperatures according to the procedures described in the Materials and Methods.

**Figure 4:**
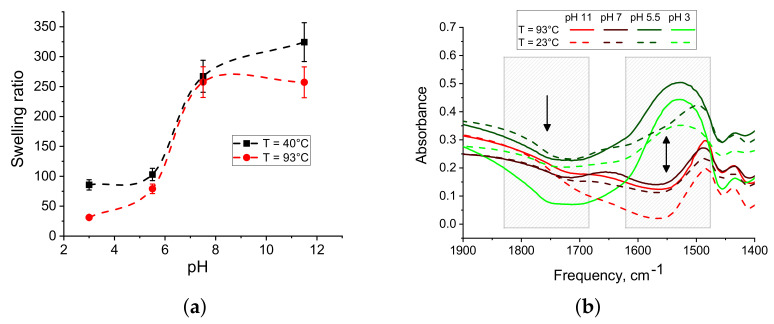
(**a**) Dependence of the equilibrium degree of swelling of PAA films on pH and annealing temperature, the lines are drawn to guide the eye. (**b**) IR spectra of linear PAA dried under different conditions.

First of all, let us consider how the pH of the solution from which PAA macromaterials were prepared and annealing temperature affects their equilibrium swelling (Figure 4a). Similar to the macromaterials formed of IPN microgels, the higher pH of the initial PAA solution the bigger the increase in the degree of swelling. In order of magnitude of the degree of swelling, two ranges of values of pH can be distinguished: below and above 6.5. Concerning the influence of annealing temperature, the higher it is, the lower the equilibrium degree of swelling. Thus, the effectiveness of interchain crosslinking is pH and temperature-dependent, which is the representation of the anhydride bond formation mechanism.

Figure 4b represents the IR-spectra of PAA macromaterials and helps to analyze the formation of anhydride bonds for the understanding of the process of crosslinking in more detail. The bands in two characteristic areas in the frequency ranges 1690–1840 cm${}^{-1}$ and 1475–1625 cm${}^{-1}$ correspond to the formation of anhydrides [39,40] and presence of COO^-^ groups [41], respectively. As it can be seen, the broadening of absorption bands characteristic to the formation of anhydrides is observed for samples obtained from solutions with low pH values; this effect is even higher for annealed samples. Apparently, the degree of dissociation of the carboxyl group R−COOH = R−COO^-^ + H^+^ affects the number of formed anhydride bonds. The efficiency of the formation of additional inter and intracrosslinks during the preparation of macromaterials should be higher in the acidic medium. This explains the difference in the degrees of swelling of macromaterials obtained from solutions and with low and high pH for both PAA and IPN-based macromaterials. It is quite expected that the bands corresponding to deprotonated carboxyl groups increase with an increase in the pH of the PAA solutions from which the macromaterial films were prepared. However, this provides an additional explanation for the greater degree of swelling of the samples of macromaterials obtained from dispersions with a higher pH.

To summarize this section, both temperature and pH of the PNIPAM-PAA IPN microgel dispersions influence strongly the properties of the prepared microstructured macrogels. This allows obtaining the materials with pre-chosen characteristics and responsive qualities. However, in experiments only, it is quite difficult to study in detail the effect of separate parameters on the final material properties. For example, in the case of PAA, the pH values change both its crosslinking ability and chain conformation. Therefore, for further analysis of these systems, we employed the tools of the computer simulations method.

### 3.2. Computer Simulations of Microstructured Macromaterials Based on IPN Microgels

The suggested scheme of computer simulations allows to setup separately the conformation of each subnetwork, the crosslinking reaction rate, and the resulting fraction of crosslinked monomers (conversion rate $c_{\mathrm{macro}}$). In turn, these parameters determine the topology of the resulting network, and, consequently, its swelling behavior. In contrast, one of the major complexities of a real-life experiment is an interconnection between all these parameters. Both pH and temperature choices affect not only subnetwork conformations, but also reaction rate and the fraction of monomers available for crosslinking, and the exact relations are unknown. Thus, we were unable to simulate experimental conditions precisely. Instead, we utilized the main advantage of the computer simulatons, investigated the individual effect of each control parameter, and suggested the explanation of experimental data based on this evidence.

**Figure 5:**
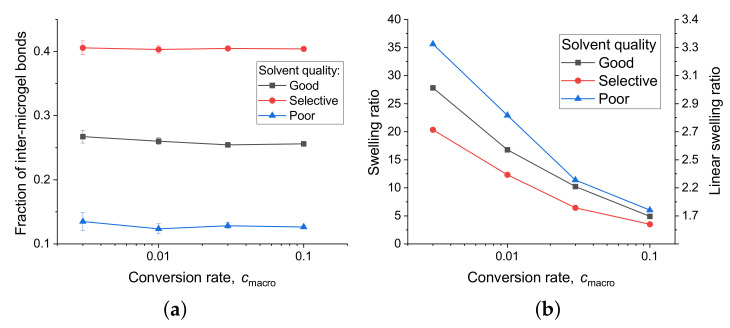
Dependence of fraction of bonds between neighbor microgels φ (**a**) and swelling or linear swelling ratio (**b**) on conversion rate for systems crosslinked in various types of solvent. Statistical errors that exceed points size are shown in bars.

**Figure 6:**
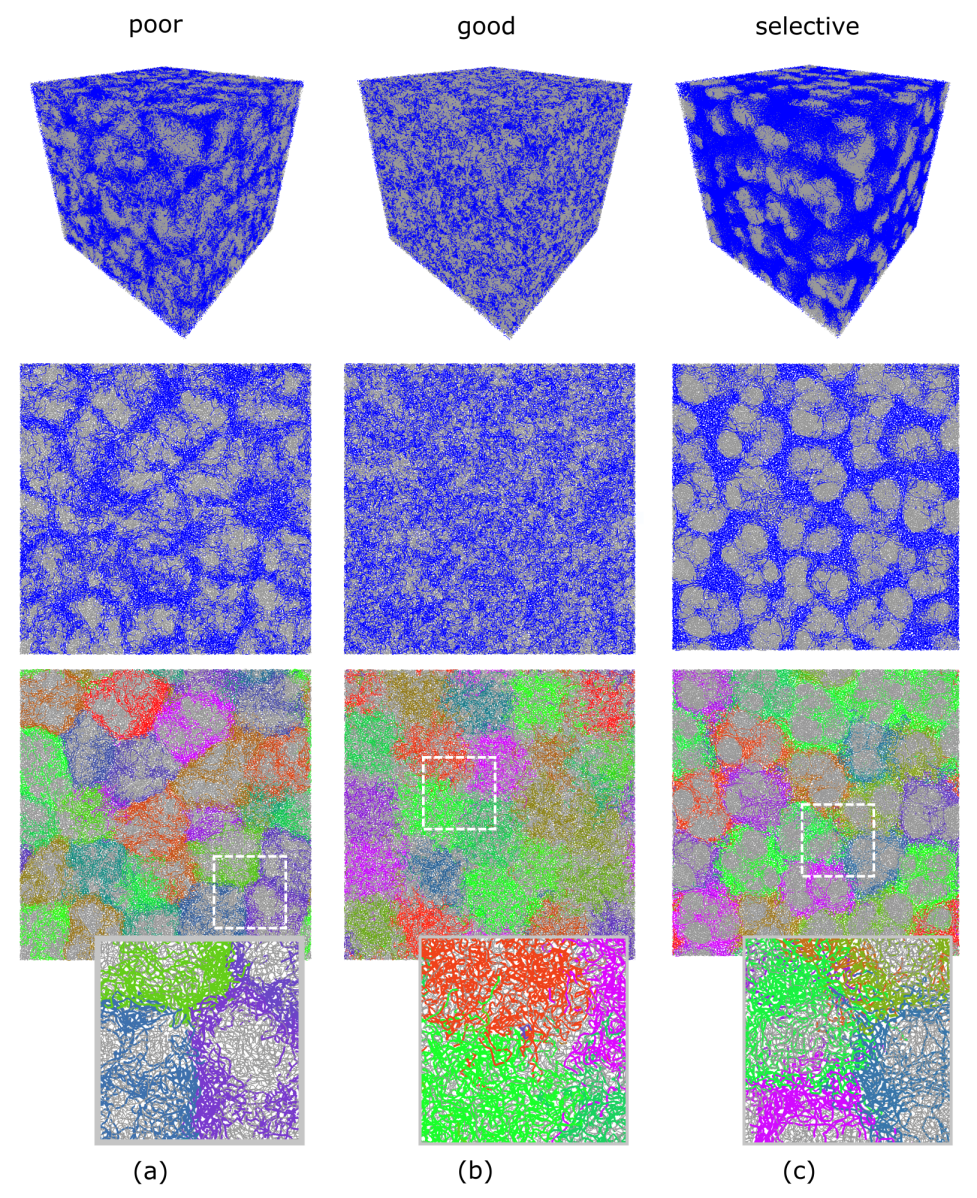
Snapshots of the simulated systems at concentration c=0.9 before crosslinking: in poor solvent (**a**), in good solvent (**b**), and in selective solvent (**c**). First and second rows are colored by non-crosslinking (gray) and crosslinking (blue) networks. In third row, non-crosslinking networks are drawn in grey, and crosslinking networks of individual microgel particles are shown in different colors.

First of all, we analyzed the topological properties of the crosslinked macrogels. For this, we divided all additional bonds between the monomers in intra-microgel additional bonds (i.e., ones formed between monomers belonging to the same microgel particle) and inter-microgel ones (i.e., bonds formed between monomers belonging to different microgel particles). Figure 5a depicts the fraction φ of inter-microgel bonds for the systems crosslinked in good, selective, and poor solvent (similar to the experimental systems at pH 7, T $=23{\;}^{\circ }$C, pH 3, T $=23{\;}^{\circ }$C, and pH 3, T $=40{\;}^{\circ }$C, correspondingly). This fraction does not depend on the conversion rate (at least, up to $c_{\mathrm{macro}}=10\%$). At the same time, it strongly depends on the subnetworks conformation. In the system before the crosslinking in a good solvent, all components are mixed (see Figure 6b), and non-crosslinking subnetworks may sterically hinder crosslinking subnetworks of different microgels from the intersection with each other, resulting in φ≈0.26. The highest fraction is reached in selective solvent (φ≈0.40), because crosslinking subnetworks expand and intersect intensively with each other, while non-crosslinking subnetworks collapse and only weakly interfere the interaction of the first ones (see Figure 6c). In contrast, poor solvent leads to the smallest fraction of inter-microgel bonds (φ≈0.13), because the collapse of subchains makes it difficult to diffuse into another microgel (see Figure 6a). It is interesting that the structure in poor solvent looks overall like the intermediate case between ones in good and selective solvents: first and second networks are moderately separated (gray and blue in the top and middle rows of Figure 6). At the same time, the detailed analysis of the level of individual microgel particles (Figure 6, bottom row) demonstrates that the crosslinking networks of individual microgels hardly interpenetrate each other in a poor solvent, while they are mixed a lot in a good and selective solvent. However, in a good solvent they are also mixed with non-crosslinking networks, which lowers the number of inter-microgel bonds, and in a selective solvent, non-crosslinking networks are totally separated.

Next, we simulated the swelling of each crosslinked system in a good solvent. For this, we changed the solvent conditions (via interaction parameters, see Materials and Methods) and applied gradual uniaxial stretching to each system. The stretching rate was equal to $5\times 10^{6}$ CG MD steps per ×4.5 increase in linear size (which is equivalent to a swelling ratio ≈100). We determined the equilibrium size of a swollen system by measuring the size of the system, at which the pressure drops to zero (pressure was averaged by 200 points calculated every 1000 CG MD steps). The swelling ratio was calculated as a ratio between the volume of a system in the swollen and the collapsed states (which is equal in our case to a number density of a system in the swollen state). The results are shown in Figure 5b. For better understanding, the scale on the right shows the linear swelling ratio which is equal to the cubic root of the swelling ratio. The observed impact of the solvent quality at the crosslinking stage on the swelling ratio: at all conversion rates, the highest swelling ratio is in systems crosslinked in a poor solvent, the lowest one is in systems crosslinked in a selective solvent, and crosslinking in a good solvent resulted in intermediate values of the swelling ratio. It is perfectly explained by the measured fraction of inter-microgel bonds, in which the higher the φ the weaker the swelling of the sample, as it indicates the higher interconnectivity between the microgel particles. At the same time, swelling ratio depends much stronger on the conversion rate $c_{\mathrm{macro}}$ (from 3.5–6 at $c_{\mathrm{macro}}=0.1$ to 20–35 at $c_{\mathrm{macro}}=0.003$).

The results of computer simulations help to qualitatively explain the obtained experimental data. First, the systems prepared at pH 3 demonstrate a much lower value of the swelling ratio compared to ones prepared at pH 7. We attribute it to the significant difference in the number of additional bonds formed during annealing (conversion rate $c_{\mathrm{macro}}$ in terms of simulations). It corresponds to the fact that the fraction of monomers that can form the anhydride bonds and, thus, serve as crosslinks, is much bigger at pH 3 values than at pH 7. From computer simulations, the difference is about 2 orders of magnitude in our case. Second, the swelling ratio of the system prepared at pH 7, $T=23{\;}^{\circ }\;$C (fully swollen IPNs) is slightly higher (+12%) in comparison to the one prepared at pH 7, $T=40{\;}^{\circ }\;$C (selective swelling). Simulations show qualitatively similar results for the systems prepared in good and selective solvents, correspondingly.

**Figure 7:**
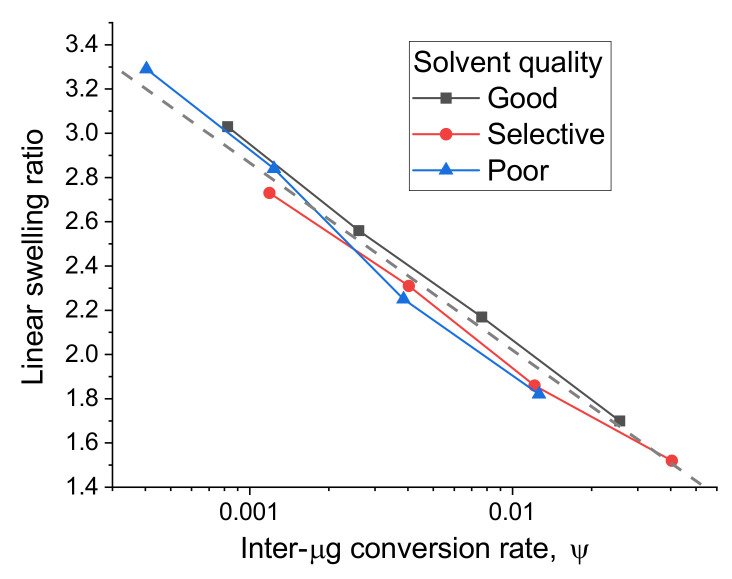
Linear swelling ratio for systems crosslinked in various types of solvent as a function of inter-microgel conversion rate. Statistical errors do not exceed the size of the points.

Finally, for a better understanding of the behavior of macrogels crosslinked from microgels, we calculated the effective conversion rate ψ calculated as the conversion rate $c_{\mathrm{macro}}$ multiplied by the fraction of inter-microgel bonds φ. This value characterizes the total interconnectivity of the particles. Then we compared the swelling ratio of all the systems by plotting it versus ψ (see Figure 7). The linear swelling ratio demonstrates near log-linear dependence on the effective conversion rate ψ. Surprisingly, the data for all systems fit into a single trend (shown by a dashed line in Figure 7), regardless of the solvent type at the crosslinking stage. The presence of such universality indicates that the swelling behavior of the studied systems depends solely on the interconnectivity of the particles (characterized by ψ) but not on other topological properties.

### 3.3. Microscopic Structure of Macromaterials

**Figure 8:**
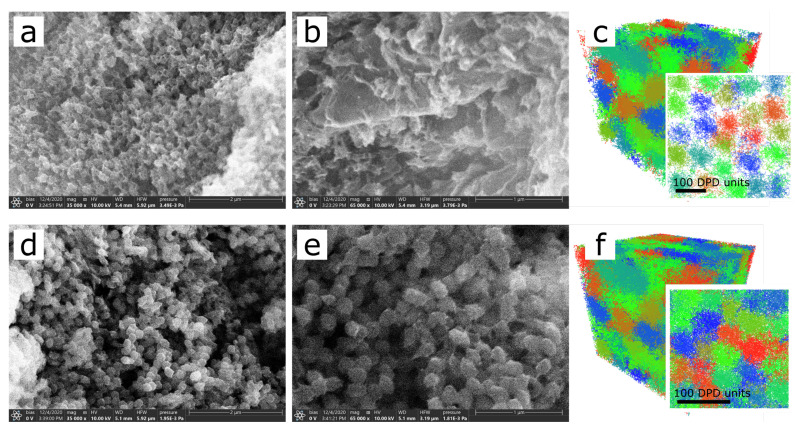
SEM microphotographs of microgel macromaterial cross-sections; (**a**, **b**) obtained at pH 7, (**d**, **e**) obtained at pH 3. Snapshots of the simulated systems with $c_{\mathrm{macro}}=0.003$ (**c**) and $c_{\mathrm{macro}}=0.1$ (**f**) after swelling, colored by individual micogel particles.

We used the scanning electron microscopy method for the further experimental investigation of the internal structure of the obtained microstructured macrogels. We acquired the images of the microgel macromaterials cross-section by studying the freshly made chipped pieces of the frozen sample. Figure 8 shows the results for the materials obtained at two different conditions—at pH 7 (Figure 8a,b), which corresponds to the smaller number of groups available for crosslinking and swollen conformation of the PAA network, and at pH 3 (Figure 8d,e), characterized by a higher amount of possible crosslinks and more compact conformation of PAA subnetwork. It could be seen that the materials obtained under higher pH conditions consist of loosely placed microgel particles connected by spikes of dried polymer chains, presumably PAA. On the contrary, materials prepared in low pH conditions (bottom row in Figure 8), are formed by densely packed and compact spherical microgel particles. These observations are in good agreement with the behavior of the materials upon swelling.

Similar results were obtained by visualization of computer simulations of materials made of crosslinked microgels. Figure 8c and f show the structure of simulated materials upon equilibrium swelling after crosslinking procedure for different conversion rates. It could be seen that the increase of crosslink amount leads to a much denser material structure, which resembles the experimental data described above.

## 4. Conclusions

PNIPAM-PAA-based macromaterials were developed by annealing of concentrated PNIPAM-PAA IPN microgel dispersions as a result of additional PAA subnetwork crosslinking due to anhydride bond formation.

The influence of the conformation of the PNIPAM-PAA IPN microgel subnetworks on the swelling behavior, pH-, and thermosensitivity and inner structure of the obtained macromaterials was investigated. The conformational states of the subnetworks were controlled by pH and pre-drying temperature before the annealing treatment.

It was found that low pH and pre-drying temperature higher than LCST for PNIPAM leads to the formation of the highest number of crosslinks between microgel particles and allows to obtain the more dense material. This is explained both by the spatial arrangement of the subchains of the polymer networks relative to each other and the influence of pH and temperature on anhydride bonds formation ability. The difference in the degree of swelling for the macromaterials prepared at high and low pH is one order of magnitude.

The thermo- and pH-sensitive properties of the obtained macromaterials are also determined by the conditions of the preparations. The materials obtained from dispersions with high pH have very low thermal sensitivity. The pH sensitivity of the macromaterials also significantly depends on the pH of PNIPAM-PAA IPN microgel dispersions.

SEM measurements revealed the difference in the microstructure of the developed macromaterials with respect to medium conditions and conformation of the PNIPAM-PAA IPN microgel subnetworks during preparation.

For the first time, the computer simulations by coarse-grained molecular dynamics method were used to mimic the process of crosslinking of IPN microgels into a single macromaterial. Computer simulation studies have confirmed that the conformational state of the microgel subnetworks and their mutual relative penetration into each other can have a significant effect on the topology and swelling coefficients of the final material.

As a result of the research carried out, the possibility to obtain polymeric materials with a controlled density, inner structure, and response to external environmental conditions were demonstrated. This opens up new opportunities for the more rational design of the experiments to produce macromaterials with the desired properties, depending on their field of application.

## Figures and Tables

**Table 1:** Swelling coefficients of macromaterials.

	pH 3, T = 23${\;}^{\circ }$C	pH 7, T = 23${\;}^{\circ }$C	pH 3, T = 40${\;}^{\circ }$C	pH 7, T = 40${\;}^{\circ }$C
$m_{\mathrm{sw}}$/$m_{\mathrm{dry}}$	2.2 ± 0.2	162 ± 16	13.2 ± 1.0	144 ± 14
$m_{T=45}$/$m_{T=25}$	0.93 ± 0.09	0.93 ± 0.09	0.30 ± 0.03	0.90 ± 0.09
$m_{\mathrm{pH}\;8}$/$m_{\mathrm{pH}\;3}$	5.5 ± 0.5	20.6 ± 2	11.2 ± 1.0	1.4 ± 0.1

## Data Availability

This study are available on request from the correspondence author.
